# T Regulatory Cells in Cord Blood—FOXP3 Demethylation as Reliable Quantitative Marker

**DOI:** 10.1371/journal.pone.0013267

**Published:** 2010-10-12

**Authors:** Jing Liu, Anna Lluis, Sabina Illi, Laura Layland, Sven Olek, Erika von Mutius, Bianca Schaub

**Affiliations:** 1 Department of Pulmonary and Allergy, University Children's Hospital Munich, Ludwig-Maximilians-Universität Munich, Munich, Germany; 2 Department of Respiratory Medicine, The Second Hospital of Ji Lin University, Chang Chun, China; 3 Institute for Medical Microbiology, Technische Universität Munich, Munich, Germany; 4 Epiontis GmbH, Berlin, Germany; New York University, United States of America

## Abstract

**Background:**

Regulatory T-cells (Tregs), characterized as CD4+CD25^hi^ T-cells expressing FOXP3, play a crucial role in controlling healthy immune development during early immune maturation. Recently, FOXP3 demethylation was suggested to be a novel marker for natural Tregs in adults. In cord blood, the role and function of Tregs and its demethylation is poorly understood. We assessed FOXP3 demethylation in cord blood in relation to previously used Treg markers such as CD4^+^CD25^hi^, FOXP3 mRNA, protein expression, and suppressive Treg function.

**Methodology:**

Cord blood mononuclear cells (CBMC) were isolated from 70 healthy neonates, stimulated for 3 days with the microbial stimulus lipid A (LpA), and allergen Dermatophagoides pteronyssinus (Derp1). Tregs (CD4+CD25^hi^, intracellular, mRNA FOXP3 expression, isolated cells), DNA methylation of the FOXP3-locus and suppressive Treg function were assessed.

**Principal Findings:**

Demethylation of FOXP3 in whole blood was specific for isolated CD4+CD25^hi^ Tregs. Demethylation of FOXP3 was positively correlated with unstimulated and LpA-stimulated FOXP3 mRNA-expression (p≤0.05), and CD4^+^CD25^hi^ T-cells (p≤0.03). Importantly, increased FOXP3 demethylation correlated with more efficient suppressive capacity of Tregs (r = 0.72, p = 0.005). Furthermore, FOXP3 demethylation was positively correlated with Th2 cytokines (IL-5, IL-13) following LpA-stimulation (p = 0.006/0.04), with Th2 and IL-17 following Derp1+LpA-stimulations (p≤0.009), but not Th1 cytokines (IFN-γ).

**Conclusions:**

FOXP3 demethylation reliable quantifies Tregs in cord blood. FOXP3 demethylation corresponds well with the suppressive potential of Tregs. The resulting strict correlation with functionally suppressive Tregs and the relative ease of measurement render it into a valuable novel marker for large field studies assessing Tregs as qualitative marker indicative of functional activity.

## Introduction

Regulatory T-cells (Tregs) are essential for the downregulation of T cell responses to both foreign and self antigens and play an important role in several immune-mediated diseases, such as allergic diseases [1]. Various studies including ours suggested that Tregs may play a crucial role in early allergy protection by keeping the immune system in balance and counter-regulating potential default pathways [2]–[4]. The general picture of Treg development is that there are two different sources of Treg, one is derived from the thymus (natural or nTreg) and one develops in the periphery (induced or iTreg), which acquire a Treg phenotype and function in the periphery [5].

In several human studies, Tregs were best characterized as CD4^+^CD25^hi^ T-cells expressing the forkhead box transcription factor (FOXP3), which suppress T cell activation and regulate various immune reactions in vitro and in vivo [6]–[8]. We and others have demonstrated that Tregs were present and functional in cord blood mononuclear cells [3]–[4], [9], though less suppressive compared to adult peripheral blood mononuclear cells (PBMC) [3].

The presence and function of Tregs in cord blood may be evolutionary useful and essential to prevent immune dysregulation resulting in either acute infections or chronic immune modulation as suggested for chronic diseases such as allergies or autoimmune disorders. For international multicenter population studies assessing Tregs for example in the development of allergic diseases in early childhood, it is critical to use a parameter for Tregs, which is specific and at the same time feasible to assess in large field studies when storage and transport conditions do not allow immediate assessment of Tregs by flow cytometry. Thus, a marker specific for Tregs and indirectly indicating effective function might provide means for reaching an improved understanding of early immune regulation of Tregs in the development of immune-mediated diseases.

FOXP3 has been shown to be the most specific marker for Tregs representing a central control element for the development and function of Tregs [10]. However, transient FOXP3 mRNA was also observed in activated non-Tregs, most notably in conventional CD4+ T-cells upon activation [11]–[12]. Thus, FOXP3 expression does not allow the unambiguous characterization of Tregs in humans. One option would be to additionally assess amount and functional capacities of Tregs by additional markers using flow cytometry and functional suppression assays [4], [13], however this is generally not feasible in large field studies. Recently, Floess S et al. described that demethylation of CpG motifs of FOXP3 was displayed especially in natural Tregs, but not or incomplete in naïve CD25^−^CD4^+^ T-cells or in vitro TGF-β-induced FOXP3^+^ Tregs [14]–[15]. They detected that stable FOXP3 expression was found only for cells displaying demethylation at a TSDR (Treg-specific demethylated region) [16]. Thus, TSDR was suggested to be an important methylation-sensitive element regulating FOXP3 gene expression. Epigenetic imprinting in this region may be critical for establishment of a stable Treg lineage. To date, TSDR demethylation has been proven to be specific for Tregs only in adults.

There are no markers reliably differentiating between natural and induced Tregs in vivo. In contrast, it appears that all in vivo Tregs (independent of their origin) express CD25 and Foxp3 and they are demethylated in the TSDR. Since iTreg are not generally expected to be present in cord blood, this tissue appears to be uniquely suitable for the characterisation of natural, thymus derived Tregs. Thus, demethylation of Tregs may be a very reliable and specific marker useful and feasible to assess in large field studies in cord blood, however not examined yet.

Therefore, we aimed to assess Treg demethylation as novel marker in cord blood in relation to previously used Treg markers such as CD4^+^CD25^hi^ surface expression, FOXP3 mRNA and protein expression, and evaluate Treg demethylation in relation to the suppressive function of Tregs on effector cells.

## Methods

Written informed consent was obtained from the mothers for participation. Approval was obtained from the local human research committee of the Bavarian Ethical Board, LMU Munich, Germany.

### Study populations

Fetal cord blood (n = 60) was obtained from a birth-cohort-study, which was performed from July 2005 to September 2008 in the outer Munich area in Germany. Pregnant mothers were recruited in the last trimester of pregnancy. Inclusion criteria comprised healthy neonates and mothers with uncomplicated pregnancy. Exclusion criteria were preterm deliveries, multiple gestation (twins, triplets), perinatal infections including fever around birth, maternal intake of medication and maternal chronic diseases. 59 samples were included with complete immunological data. Additional donors (n = 5) were recruited for isolation experiments by random selection procedure.

### FOXP3-specific real-time PCR

Genomic DNA was isolated from EDTA-handled cord blood with DNeasy Blood-Kit (Qiagen, Hilden, Germany). From 2ml EDTA-blood, a recovery of 300–900 µg/µl DNA is sufficient for the 20ng of DNA, which is required for methylation analysis. Real-time-PCR for measurement of the FOXP3 Treg-specific demethylated region (TSDR) was performed in 20µl using Roche LightCycler® 480 Probes Master (Roche Diagnostics, Mannheim, Germany) containing 15pmol of methylation or non-methylation-specific forward and reverse primers for TSDR, 5pmol hydrolysis probe, 200ng lambda-DNA (New England Biolabs, Frankfurt, Germany) and 30ng bisulfite-treated genomic DNA template or respective amount of plasmid standard [16]. Each sample was analyzed in triplicate using a Light-Cycler 480 System. Since Foxp3 is X-linked, in all male Treg cells the one Foxp3 gene is totally demethylated. In females, having two X-chromosomes, in Treg cells X-inactivation leads to complete inactivation and methylation of the bar body and one out of the two X-chromosomes in female Treg cells remains fully methylated. The other “open” x-chromosome is demethylated in female Tregs. For calculation of Treg ratios in a given cell sample, it is necessary to multiply with a factor of 2 in order to determine the Treg number. This has been done in all female samples.

### Isolation and culture of CBMC

Cord blood mononuclear cells (CBMC) were isolated by density-gradient centrifugation with Ficoll-Hypaque. CBMC were stimulated with lipid A (LpA, 0.1µg/mL), phytohemagglutinin (PHA, 5µg/mL), and in addition Dermatophagoides pteronyssinus (Derp1, 30µg/mL) for 3 days as previously described [4] and compared to unstimulated samples. Significant changes through endotoxin were excluded by functional assays as previously described [17]. Cytokines were measured in supernatants; cells were collected for mRNA extraction or flow cytometry.

### Real-time quantitative RT-PCR

Total RNA was isolated from CBMC with TRI Reagent, and reverse transcribed to cDNA with reverse transcriptase (Invitrogen, Karlsruhe, Germany). Specific primer pairs for 18-S, FOXP3 were designed with Vector NTI advance10 (Invitrogen, Karlsruhe, Germany) as described before [17]. Direct detection of the PCR product was monitored by measuring the increase in fluorescence caused by the binding of SYBR Green to dsDNA using iCycler (BIORAD, Munich, Germany). The determined threshold cycle (ct) is the number of PCR cycles required for the fluorescence signal to exceed the detection threshold, which was set to the log-linear range of the amplification curve. Lower delta CT represents higher mRNA expression.

### Flow cytometry

CBMC were analyzed using 3-color flow cytometry (FACScan, Becton-Dickinson, Germany). For surface staining, 2µl anti-human CD4-FITC, 1µl CD25-PC5 (Immunotech, Beckman Coulter, Marseille, France), 1µl IgG1-FITC (Dako Cytomation, Denmark), 0.5µl IgG2a PC5 (BD Pharmingen, Germany) was added. For intracellular FOXP3 staining, 8 µl anti-human CD4-FITC, 4µl anti-human CD25-PC5 antibodies (1×10^6^/100µl) were used, cell permeabilization was performed, and 10µl FOXP3-PE/corresponding isotype-control antibodies (Natutec eBioscience, Munich, Germany) was added. Samples were measured with FACScan (Becton Dickinson, Heidelberg, Germany). Data were analyzed with CellQuest software (Becton Dickinson, Germany), post acquisition analysis was performed with WinMDI 2.8 software (Becton Dickinson, Mountain View, CA, USA).

### Isolation and suppressive function of Tregs

APCs were isolated after positive depletion of CD3^+^ cells (CD3 isolation-kit, Miltenyi Biotec, Germany) and irradiated (30 Gy, 3000 rad, 10 min). CD4^+^CD25^−^ effector T-cells and CD4^+^CD25^+^T-cells were isolated (two-step-procedure), using depletion of non-CD4+cells, followed by positive selection of CD4^+^CD25^+^T-cells (Miltenyi Biotec, Germany). CD4^+^CD25^−^ T-cells (2×10^4^/well), labelled with 5µM CFSE (Invitrogen, Germany) were incubated for 3 days with irradiated CD3- cells (4×10^4^/well) in coculture with/without CD4^+^CD25^+^T-cells (2×10^4^/well) with 0.8µg/ml PHA stimulation. Division and proliferation of CD4^+^CD25^−^ T-cells were assessed by flow cytometry or 3[H]-thymidin incorporation, respectively. The suppressive capacity of Tregs was defined as the following:
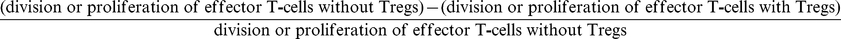



### FACS Sorting

CBMC were stained using the surface markers anti-human CD4-FITC (40µl) and CD25-PC5 (20µl, Immunotech, Beckman Coulter, Marseille, France) per 1×10^7^ cells and sorted by FACS (MoFlo XDP, Dako) in two subpopulations: CD4^+^CD25^−^ and CD4^+^CD25^hi^. DNA extraction was performed with QIAamp DNA Micro-Kit (Qiagen, Hilden, Germany) and FOXP3-methylation specific real-time PCR as described above.

### Cytokine measurements

The concentrations of cytokines in supernatants were measured after 3 days of stimulation with the Human Cytokine Multiplex Assay Kit according to the manufacturer's instructions, Bio-Rad, Munich, Germany). The limits of detection (pg/ml) were 1.8 (IL-5), 2.1 (IL-13), 0.2 (IL-17) and 1.3 (IFN-γ). Non-detectable cytokine concentrations were assigned to a value of 0.01 for inclusion into the analysis.

### Statistical analysis

Data analysis was performed with SigmaStat3.5, SPSS14.0 and SAS 9.2 (SAS-Institute, Inc. Chicago, Ill) software. Correlations were analyzed using Pearson Product Moment Correlation or Spearman Rank Order Correlation based on distribution of the data. T-test was used for the comparison of group differences of suppressive capacity of Tregs (%). Statistical significance was defined by p<0.05.

## Results

### Specificity of FOXP3 demethylation for isolated Tregs in cord blood

**Figure 1:**
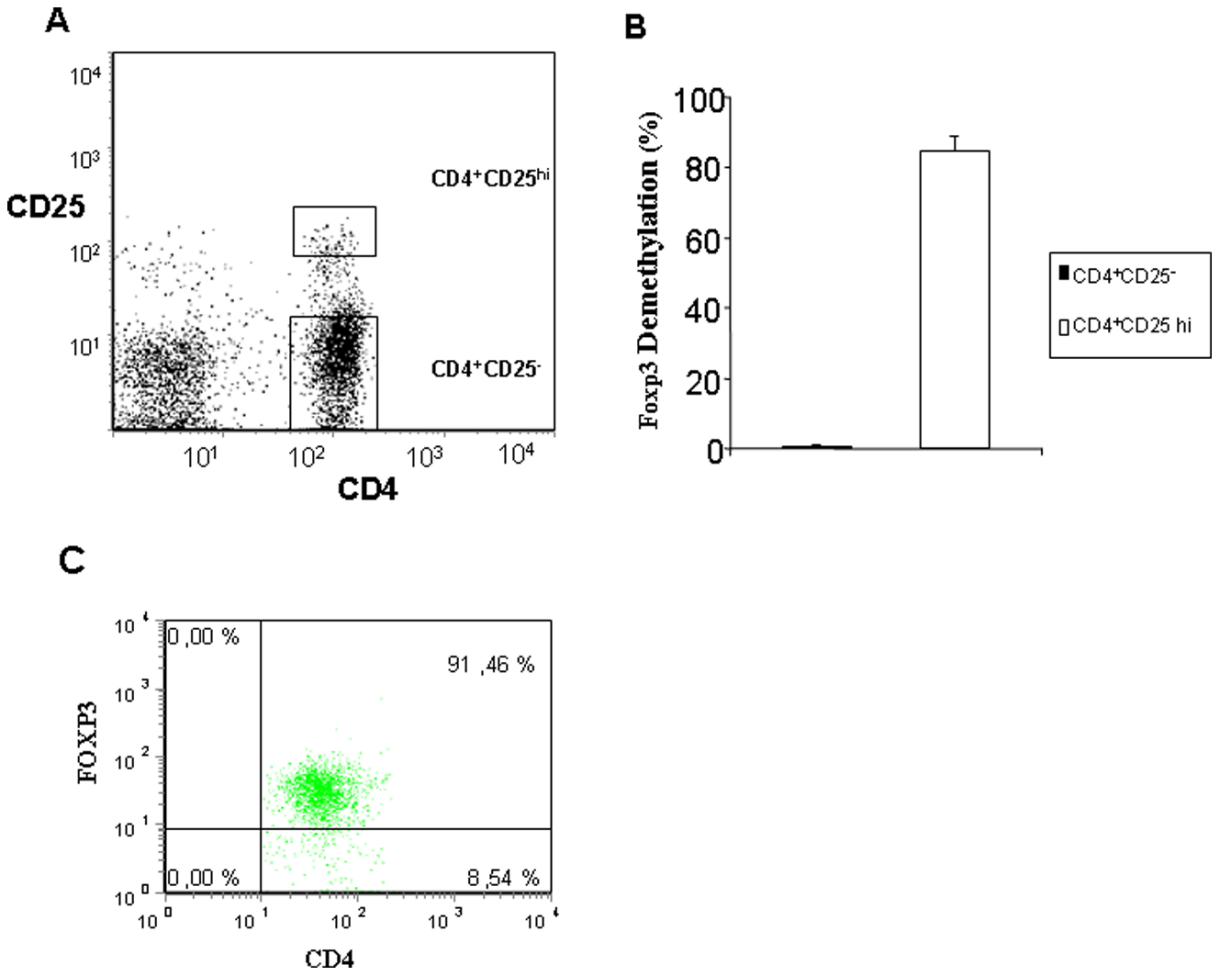
FOXP3 demethylation in isolated CD4CD25^−^ and CD4CD25^hi^ cells. A/B. CD4CD25^−^ and CD4CD25^hi^ cells were isolated with Dako MoFlow. DNA was extracted and demethylation (%) of FOXP3 was measured with real-time PCR. C. Intracellular FOXP3 protein was measured in CD4CD25^hi^ cells as described in methods. N = 5, mean ± SEM were shown in B, one representative sample for A/C.

In FACS-sorted isolated CD4^+^CD25^−^ T-cells and CD4^+^CD25^hi^ T-cells from cord blood, FOXP3 demethylation was hardly detectable in CD4^+^CD25^−^ cells and well detectable in a mean of 81% in the CD4^+^CD25^high^ T cells (Fig. 1A,B). In parallel, Foxp3 protein was expressed intracellular in a mean of 90% for CD4^+^CD25^hi^ T-cells; one representative sample is shown in Fig. 1 C.

### Demethylation of FOXP3 in whole blood was positively correlated with unstimulated and LpA-activated FOXP3 mRNA expression in cord blood

**Figure 2:**
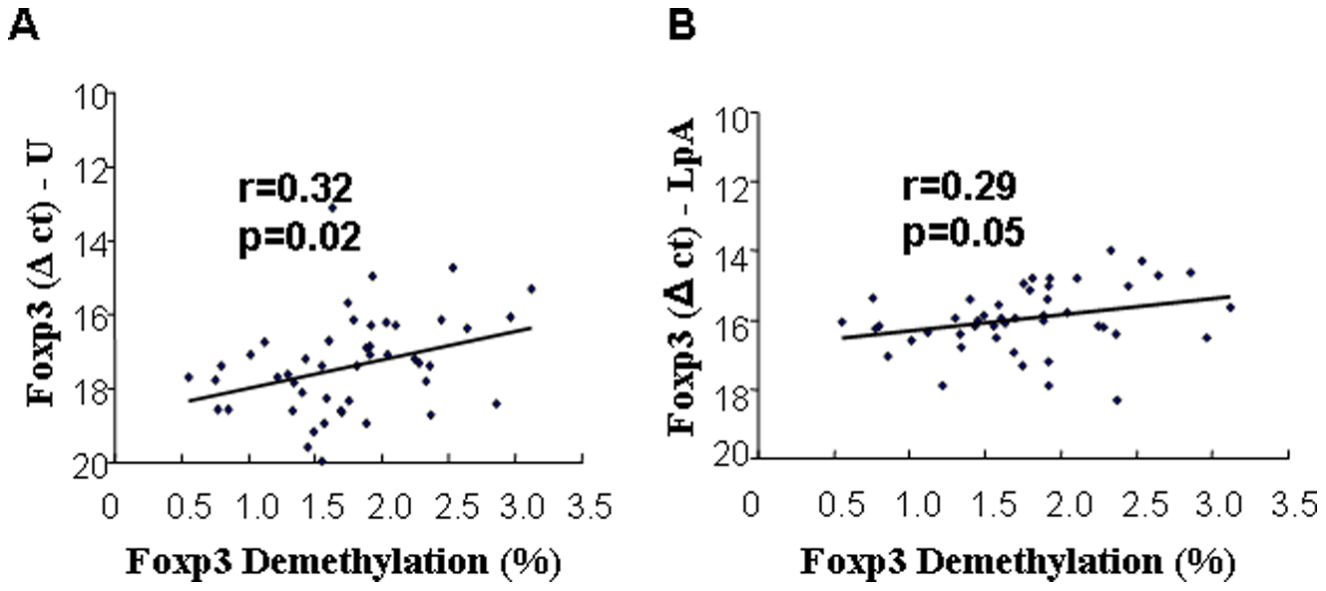
Correlation between demethylation of FOXP3 and unstimulated and LpA-activated FOXP3 mRNA expression. Demethylation (%) of FOXP3 in whole cord blood was measured with real-time PCR. FOXP3 mRNA expression was measured with real-time RT-PCR in unstimulated (A) and LpA-stimulated CBMCs (B), which were shown as delta ct to 18S. Lower delta ct represents higher mRNA expression. N = 48; correlation was analyzed with Pearsons correlation coefficient.

FOXP3 mRNA expression is currently used in several studies assessing Tregs, in particular in the context of microbial exposure such as Lipid A, LpA (TLR4 stimulus). In Fig. 2, FOXP3 mRNA expression is shown as delta ct values. A lower delta ct corresponds to higher FOXP3 gene expression. FOXP3 mRNA expression in unstimulated and LpA-stimulated cells was positively correlated with demethylation (%) of FOXP3 in whole blood, though with low correlation coefficient (r = 0.32, p = 0.02; r = 0.29, p = 0.05, Fig. 2A/B). Assessing stimulation with the allergen house dust mite Derp1 alone and a “natural environmental stimuli”, such as a combination of Derp1+LpA stimulation, FOXP3 mRNA expression showed no significant correlation with demethylation in whole blood (%) of FOXP3 (data not shown).

### Demethylation of FOXP3 was positively correlated with the amount of unstimulated and LpA-activated CD4^+^CD25^hi^ T-cells in cord blood

**Figure 3:**
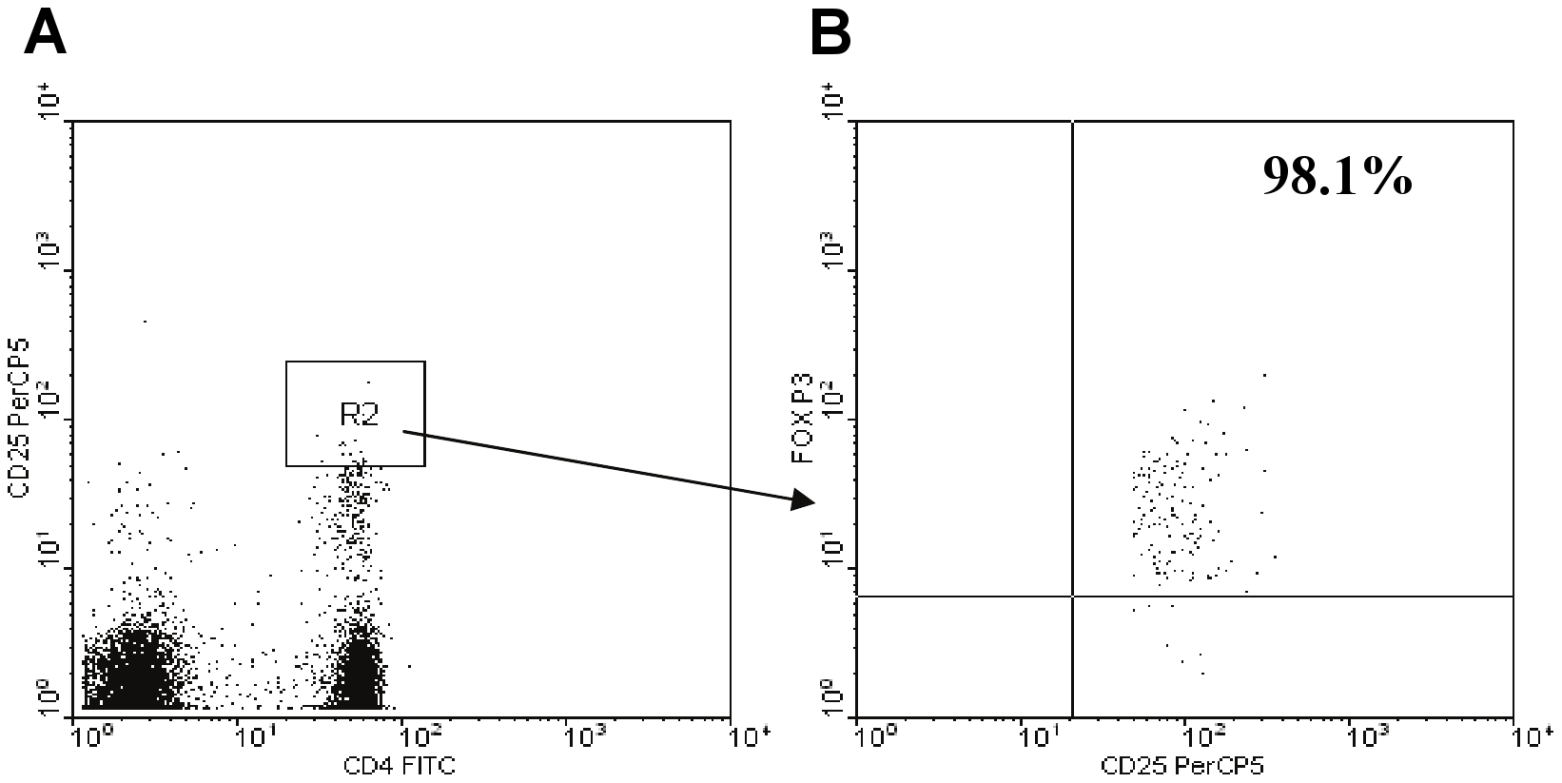
Percentage of FOXP3^+^ T-cells in unstimulated CD4^+^CD25^hi^ T-cells. Flow cytometry of unstimulated CD4^+^CD25^+^ cells as described in methods. **A**. R2 = CD4^+^CD25^hi^ T-cell gate. **B**. Out of CD4^+^CD25^hi^ T-cells, 98.1% were FOXP3^+^ T-cells.

**Figure 4:**
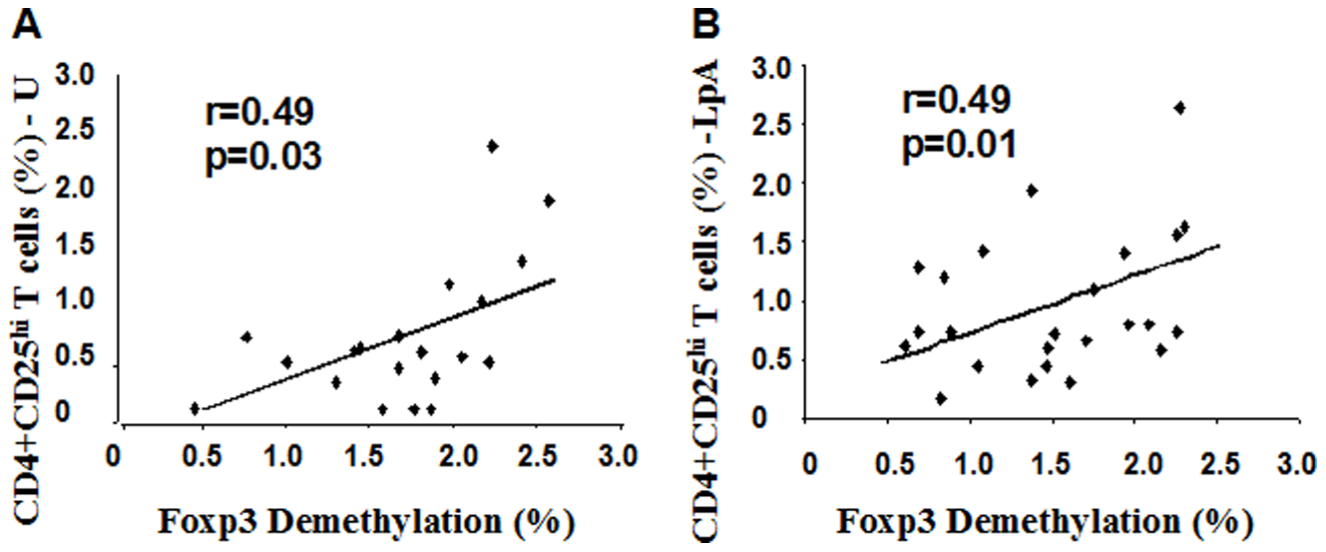
Correlation between demethylation of FOXP3 and CD4^+^CD25^hi^ T-cells (%). Demethylation (%) of FOXP3 was measured with real-time PCR. CD4^+^CD25^hi^ T-cells (% in lymphocytes) were measured with flow cytometry. **A**. Correlation between FOXP3 demethylation and unstimulated CD4^+^CD25^hi^ T-cells (%). **B**. Correlation between FOXP3 demethylation and LpA-activated CD4^+^CD25^hi^ T-cells (%). N = 20/24; correlation was analyzed with Pearsons correlation coefficient.

Tregs are commonly characterized by expression of the surface marker CD4^+^CD25^hi^. In this study, more than 98% of CD4^+^CD25^hi^ T-cells expressed FOXP3 protein in cord blood in non-sorted cells (Fig. 3). The amount of unstimulated and LpA-stimulated CD4^+^CD25^hi^ T-cells was positively correlated with increased FOXP3 demethylation (%) (Fig. 4A/B).

### Demethylation of FOXP3 was positively correlated with the suppressive capacity of Tregs in cord blood

**Figure 5:**
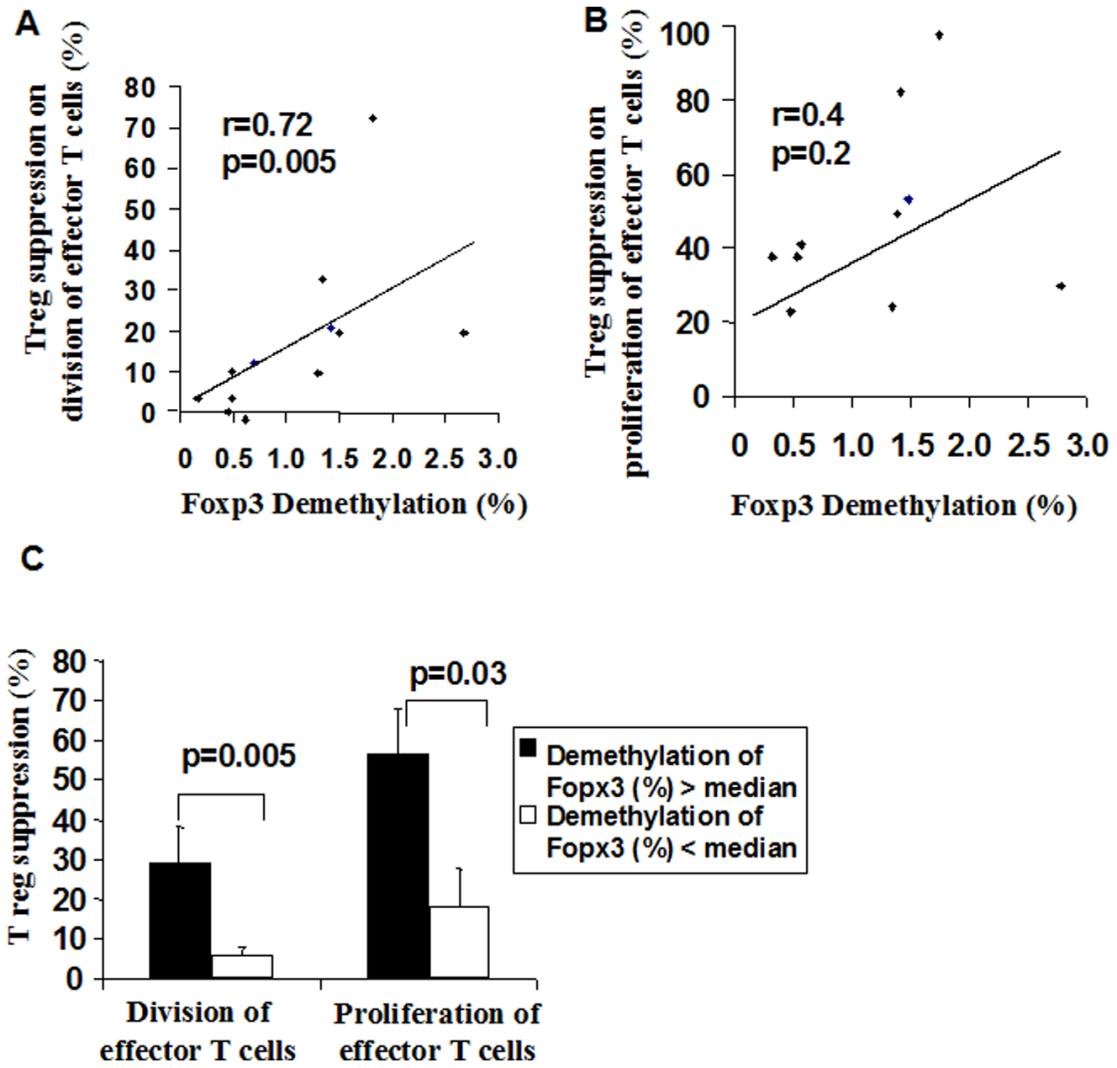
Relationship between demethylation (%) of FOXP3 and suppressive capacity of Tregs. Demethylation (%) of FOXP3 was measured with real-time PCR. **A**. The correlation of demethylation of FOXP3 and suppressive capacity of CD4^+^CD25^+^ T-cells on the division of CFSE-labelled CD4^+^CD25^−^ T effector cells. Assessment by flow cytometry. **B**. Correlation of demethylation of FOXP3 and suppressive capacity of CD4+CD25+Tcells on the proliferation of CD4^+^CD25^−^ T effector cells, measured with 3[H]-thymidin incorporation. **C**. The suppressive capacity of Tregs was more effective in cord blood with higher FOXP3 demethylation (%) (>median of 0.81). **A–B**: n = 13/12; Pearsons correlation coefficient. **C**: n = 13, t-test, data were shown as mean+-SEM.

As the amount of Tregs is not indicative of its functional regulation, we additionally assessed the suppressive capacity of CD4^+^CD25^+^ T-cells on the division and proliferation of CD4^+^CD25^−^ T-effector cells in a subgroup (n = 13). Cell division is a specific indicator of differences in cell cyles and was measured with CFSE staining of effector cells, while proliferation assessed overall capacity of effector cell proliferation measured by 3 [H]-thymidin incorporation. The suppressive capacity of cell division was positively correlated with demethylation of FOXP3 (r = 0.72, p = 0.005, Fig. 5A), while the suppressive capacity of Tregs on proliferation showed a trend with demethylation (r = 0.4, p = 0.2, Fig. 5B, n = 13). In parallel, when dividing FOXP3 demethylation according to the median (0.70%) into groups with relatively higher or lower demethylation, the suppressive capacity of Tregs on effector cells (division and proliferation) was significantly more effective in the higher compared to the lower group (p = 0.005, p = 0.03; Fig. 5C).

### The relationship between demethylation of FOXP3 and cytokine secretion in cord blood

As Tregs balance immune responses of different T cell lineages, we assessed whether demethylation of FOXP3 had an impact on effector cell responses such as Th1/Th2 and Th17 cytokine secretion. Distribution of cytokine expression (in pg/ml, median±25/75. perc) were as follows: IFN-g M 0,04 (0,01/1,03), LpA 50,35 (16,10/96,08), Derp1+LpA 70,49 (51,69/113,34); IL-5 M 0,01 (0,01/0,09); LpA 4,99 (IQR 2,17, 10,18), Derp1+LpA 7,86 (3,55/17,68); IL-13 M 0,33 (0,06/0,76), LpA 3,23 (1,46/8,22), D+L 6,16 (2,60/14,38) and IL-17 M 0,01 (0,01/0,43), LpA 1,43 (0,08/3,95), Derp1+LpA 2,82 (0,46/6,03). No significant correlation was found between FOXP3 demethylation (%) with cytokines before stimulation (Table 1). FOXP3 demethylation was positively, however not highly correlated with Th2 cytokines (IL-5 and IL-13) following LpA-stimulation. Following Derp1+LpA stimulation the correlation was stronger. Control Derp1 stimulation alone showed no significant correlation. In addition, FOXP3 demethylation was correlated with IL-17 secretion following Derp1+LpA stimulated (Table 1). There was no correlation with Th1 cytokines (IFN-γ, Table 1) and not with IL-10, IL-15 and TNF-α (not shown). IL-2 was correlated with FOXP3 demethylation after Derp1+LpA stimulation (r = 0.35, p = 0.05, n = 30).

**Table 1:** Correlation between FOXP3 demethylation (%) and cytokine production in CBMC.

Cytokines	Unstimulated		LpA stimulated		Derp1+LpA stimulated	
	r	p	r	p	r	p
**IFN-γ**	0.03	0.81	0.09	0.48	0.09	0.62
**IL-5**	0.07	0.57	0.37	**0.006**	0.55	**0.002**
**IL-13**	0.16	0.23	0.28	**0.04**	0.52	**0.004**
**IL-17**	0.07	0.59	0.09	0.52	0.48	**0.009**

n = 60. Data were analyzed with Spearman Rank Order Correlation. r = correlation coefficient.

## Discussion

We assessed quantitative and qualitative markers for Tregs in cord blood, including CD4^+^CD25^hi^ T-cells, FOXP3 mRNA and protein expression, isolated Tregs and the suppressive capacity of Tregs in relation to demethylation of FOXP3 (%) as novel marker in whole blood. Demethylation of FOXP3 was shown to be specific for isolated CD4^+^CD25^hi^ Tregs in cord blood. Demethylation of FOXP3 was positively correlated with unstimulated and trendwise for LpA-activated CD4^+^CD25^hi^ T-cells and at low levels with FOXP3 mRNA expression. Additionally, FOXP3 demethylation was positively associated with the suppressive function of Tregs on effector cell division.

Tregs are crucial T-cells in immune homeostasis, as they balance the immune system to avoid immune-mediated diseases such as allergies, asthma, autoimmune disorders, cancer, infection or graft-versus-host diseases [18]–[20]. To date, multiple markers are used to assess Tregs, including FOXP3, CD25^hi^, TGF-β, GITR, LAG3, CTLA-4, CD69, CD44, CD127-/low and CD49d [21]–[22]. The transcription factor FOXP3 is the most specific and widely used molecular marker for identification and characterization of Tregs [23]. Recently, a new marker for Tregs was described to be reliable and objective for the identification and quantification of Tregs in adults. Floess at al. showed that DNA demethylation at the FOXP3 locus (TSDR), both in mice and human adults, coincides with the generation of stable Tregs [14]–[16].

FOXP3 demethylation may be a technically precise marker in addition to currently assessed CD4^+^CD25^hi^ T-cells measurements, which is not completely standardized in particular under different stimulation conditions in large population studies. Additionally, human Tregs were shown to be a rapidly proliferating population compared to naïve or memory T-cells, but were short-lived and underwent apoptosis at a far higher rate than naive or memory cells. FOXP3 demethylation (%) may represent an intrinsically more stable parameter for Tregs than mRNA expression or protein synthesis, and can be accurately quantified [24]–[25]. However, all current data derive from murine studies or adults, where immune maturation has taken place and Tregs are often stimulated and have a different phenotype than early in life.

This study shows for the first time that early in life, namely in cord blood, FOXP3 demethylation is very low in CD4^+^CD25^−^ T cells and high in CD4^+^CD25^hi^ T-cells. Thus, from early in life, FOXP3 methylation seems to be a stable parameter for Treg assessment. While in adults the signal derives from iTregs and nTregs, we can claim that in cord blood it comes from nTregs only.

Additionally, FOXP3 demethylation was well correlated with CD4^+^CD25^hi^ expression but rather low with FOXP3 mRNA in unstimulated conditions. The latter is not surprising as FOXP3 mRNA was assessed in bulk culture. Thus FOXP3 expression may have also included expression in non-Tregs. When comparing FOXP3 demethylation with LpA-stimulated FOXP3 mRNA expression and CD4CD25^+^ high T-cells, correlations were low or showed only a trend. The rationale to use LpA-stimulation was to assess one “master stimulus” representing microbial exposure (TLR4-stimulation) as “protective stimulus” against allergy development. This shall provide the basis using FOXP3 methylation for in vivo microbial exposure in future field studies assessing childhood pulmonary diseases such as allergic asthma. The low correlations may be explained by a transient increase in CD25 and Foxp3 expression following LpA stimulation, but no change in demethylation, thus resulting in a weaker correlation.

Furthermore, assessing FOXP3 demethylation may indirectly contribute to assess Tregs with functional suppression. It is controversial whether all activated FOXP3^+^ T-cells have similar suppressive capability [26]–[28]. Thus FOXP3 demethylation (%) may contribute to distinguish natural Tregs (with complete suppressive capacity) as compared to e.g. TGF-β induced FOXP3-expressing Tregs (with incomplete suppressive capacity). In the presented study, Treg function in cord blood – representing nTregs only- was positively and strongly correlated with FOXP3 demethylation.

Thus, examining FOXP3 demethylation could potentially be applied as an indirect surrogate marker of suppressive capacity when more extensive evaluation can not be performed in large population studies - either due to technical or logistic possibilities.

As regulatory T-cells are keeping immune responses in balance, T-cell cytokines may be equally regulated by the demethylation status of Tregs. In cord blood, FOXP3 demethylation was positively correlated with Th2 cytokines (IL-5 and IL-13), however at low levels. Following Derp1+LpA stimulation, besides Th2 cytokines, also IL-17 production, but not Th1 cytokines (IFN-γ), was positively correlated with FOXP3 demethylation. A correlation of Th2-cells and IL-17 were described in two cord blood populations before [4], [17], however not related to Tregs. This effect may be due to the immature nature of cord blood representing a more Th2-shifted immune phenotype [29], and thus not completely balanced at this stage. Previously, Th2 cytokines IL-4 and IL-6 have been shown to inhibit FOXP3 induction [30]; however IL-4 can on the other hand induce proliferation of CD4CD25^+^ cells [31], thus a potential synergistic mechanism is possible. While these results derived from murine studies, cord blood is unique in terms of Treg regulations. Although in humans Tregs can inhibit cytokine production of effector cells in *in vitro* experiments [3], Tregs increased in parallel with Th2 cytokines in autoimmune pancreato-cholangitis [32] and the percentage of CD4^+^CD25^int^/CD4 T-cells was correlated positively with IL-5, IL-10, and IL-13 in pollen-sensitized children [33]. Th2 cytokines (IL-4, IL-13) can also induce FOXP3-expressing CD4^+^CD25^+^ regulatory T-cells from healthy human CD4^+^CD25^−^ precursors [34]. Moreover, Tregs can differentiate to IL-17 producing cells under specific stimulation conditions [35]–[36]. Thus, complex competitory mechanism among different Th subgroups may be involved. Following diverse stimulations, the interplay of Tregs and other Th lineages may be different, but this clearly needs to be assessed in more detail early in life.

Overall, this novel method was shown to be quantitatively comparable to previously used methods, but may be superior as an adequate tool for routine applications in large international field trials as it is technically less demanding and laborious than previously used methods [25].

In summary, FOXP3 demethylation (%) was demonstrated to be strongly correlated with Tregs early in life. FOXP3 demethylation was positively correlated with CD4^+^CD25^hi^ T-cells and to a lower degree with FOXP3 mRNA expression in cord blood. Moreover, FOXP3 demethylation may represent an indirect marker for efficient suppressive capacity of Tregs. Thus, FOXP3 demethylation may be used as a good quantitative and indirect qualitative marker and valid method in multicenter field studies, offering the possibility of an additional or potentially substitute marker for assessment of Tregs.
